# Stigmasterol-Stabilized Nanoliposomes Enhance Oral Delivery of Collagen Peptides Through Improved Systemic Exposure of Hydroxyproline-Containing Peptides

**DOI:** 10.3390/md24080293

**Published:** 2026-08-21

**Authors:** Zifang Zhao, Wenshuo Xing, Meichao Zhang, Chengsuo Liu, Weijie Zhang, Haohao Wu, Changhu Xue

**Affiliations:** 1 State Key Laboratory of Marine Food Processing & Safety Control, College of Food Science and Engineering, Ocean University of China, Qingdao 266000, China; 2 Haikou Research & Development Center for Biopeptide Engineering, Huayan Collagen Technology Co., Ltd., Haikou 570100, China; 3 Sanya Oceanographic Institution, Ocean University of China, Sanya 572024, China; xingwenshuo@stu.ouc.edu.cn (W.X.);; 4 Weihai Institute for Food and Drug Control, Weihai 264200, China

**Keywords:** collagen peptides, nanoliposomes, gastrointestinal digestion, peptide-bound hydroxyproline, oral bioavailability

## Abstract

Collagen peptides (CPs) possess diverse biological activities, yet their oral efficacy is limited by gastrointestinal degradation and restricted systemic exposure of intact bioactive peptide species. Herein, a stable cholesterol-free nanoliposome system was developed using soybean phospholipids and stigmasterol through high-pressure microfluidization, followed by tangential flow filtration and spray drying to obtain a stable dry formulation. The optimized nanoliposomes exhibited a particle size below 100 nm, high peptide loading, excellent redispersibility, and remarkable physicochemical stability during refrigerated storage and under different pH and thermal conditions. During simulated gastrointestinal digestion, the stigmasterol-stabilized phospholipid bilayer effectively preserved encapsulated CPs throughout the gastric phase while facilitating peptide release under intestinal conditions. Oral administration in rats significantly enhanced collagen peptide bioavailability, increasing the plasma exposure (iAUC_0–8 h_) of total hydroxyproline by 3.84-fold compared with free CPs. Peptide-bound hydroxyproline exposure increased 9.3-fold and accounted for approximately 94% of total absorbed hydroxyproline. UHPLC–HRMS analysis confirmed substantially enhanced systemic exposure of multiple characteristic hydroxyproline-containing dipeptides and tripeptides following nanoliposomal delivery. These findings indicate that stigmasterol-containing nanoliposomes improve the gastrointestinal stability and systemic delivery performance of collagen peptides, providing a promising strategy for enhancing the oral delivery potential of food-derived bioactive peptides.

## 1. Introduction

Collagen peptides (CPs) have attracted considerable attention as functional food ingredients owing to their diverse biological activities, including skin health promotion, bone metabolism regulation, joint protection, and tissue repair [1,2]. Unlike intact collagen, CPs possess lower molecular weights and improved water solubility, enabling efficient gastrointestinal digestion and absorption following oral administration [3]. Increasing evidence suggests that hydroxyproline-containing oligopeptides, particularly dipeptides and tripeptides such as Gly–Pro–Hyp and Pro–Hyp, can be absorbed into the bloodstream and exert physiological functions in peripheral tissues [4]. Consequently, CPs have become one of the most widely consumed bioactive protein ingredients in the nutraceutical and functional food industries.

Despite their promising bioactivities, the oral efficacy of CPs remains limited by several physiological barriers. Following ingestion, CPs are exposed to extensive enzymatic hydrolysis in the gastrointestinal tract, resulting in the generation of free amino acids and small peptide fragments [5]. Although part of the released hydroxyproline-containing peptides can survive digestion and enter systemic circulation, a substantial proportion is degraded before reaching target tissues [6]. Moreover, conventional peptide absorption mainly relies on transporter-mediated uptake pathways, which may become saturated under high-dose administration [7]. Therefore, improving the gastrointestinal stability, absorption efficiency, and systemic delivery of bioactive collagen-derived oligopeptides remains a critical challenge for the development of high-performance CPs.

Nanoliposomes have emerged as promising oral delivery systems for bioactive compounds due to their excellent biocompatibility, structural similarity to biological membranes, and ability to encapsulate both hydrophilic and hydrophobic molecules [8,9]. Recent studies have demonstrated that peptide-loaded nanoliposomes can improve peptide stability, gastrointestinal resistance, and bioaccessibility [10,11,12]. However, these studies have mainly focused on physicochemical characterization, in vitro digestion behavior, or preservation of biological activity, while the systemic fate and molecular forms of absorbed peptides after oral nanoliposomal delivery remain largely unexplored.

The application of nanoliposomes for collagen peptide delivery remains particularly challenging because CPs are highly water-soluble and readily diffuse out of the aqueous core during liposome formation and storage [13]. Unlike hydrophobic small-molecule cargos, highly water-soluble collagen peptides are difficult to retain within lipid vesicles, and their encapsulation performance is strongly influenced by peptide concentration during vesicle formation. Furthermore, most existing studies have focused primarily on encapsulation efficiency or in vitro release behavior, whereas cholesterol-free sterol regulation strategies and their influence on collagen peptide retention have been rarely investigated.

In addition to delivery efficiency, the biological significance of absorbed CPs depends strongly on their molecular form in circulation. Hydroxyproline is rarely found in conventional dietary proteins and therefore serves as a specific biomarker for tracing collagen peptide absorption [14]. More importantly, plasma hydroxyproline exists as both free hydroxyproline and peptide-bound hydroxyproline, the latter mainly representing hydroxyproline-containing oligopeptides that are considered the principal bioactive forms of CPs. Consequently, distinguishing between free and peptide-bound hydroxyproline provides a unique opportunity to evaluate not only the quantity but also the quality of collagen peptide absorption [15].

In this study, a stable stigmasterol-stabilized collagen peptide nanoliposome system was developed using soybean phospholipids and stigmasterol. Following optimization of formulation and fabrication by high-pressure microfluidization, tangential flow filtration, and spray drying, the physicochemical stability, gastrointestinal fate, and peptide retention behavior of the nanoliposomes were systematically investigated. Their influence on collagen peptide absorption was further evaluated through plasma pharmacokinetic analysis of total, free, and peptide-bound hydroxyproline, complemented by UHPLC–HRMS quantification of characteristic hydroxyproline-containing di- and tripeptides. This study establishes an efficient oral delivery platform for CPs.

## 2. Results and Discussion

### 2.1. Selection of Preparation Strategy and Lipid Composition for CP-Loaded Nanoliposomes

Due to their low molecular weight and high aqueous solubility, CPs readily diffuse across the aqueous phase during liposome formation, making efficient retention within liposomal vesicles considerably more challenging than for hydrophobic bioactives or macromolecular proteins [16]. Consequently, both the fabrication process and membrane composition are critical determinants of peptide loading and colloidal stability. To identify an appropriate preparation strategy, membrane extrusion and high-pressure microfluidization were first compared using phospholipid-to-peptide mass ratios ranging from 1:2 to 1:50 (Table 1).

High-pressure microfluidization consistently produced substantially smaller nanoliposomes with higher encapsulation efficiencies than membrane extrusion across the entire formulation range. The intense shear, cavitation, and impact forces generated within the interaction chamber rapidly disrupted multilamellar vesicles and promoted their reassembly into uniformly dispersed nanosized liposomes, thereby facilitating peptide retention during vesicle formation [17]. As peptide loading increased, particle size gradually increased for both preparation methods, reflecting the increasing difficulty of retaining highly water-soluble peptides within the aqueous core. Nevertheless, the increase was considerably less pronounced following microfluidization, indicating that rapid membrane reorganization enabled the formation of stable nanoscale vesicles even under high peptide-loading conditions. Consistent with these observations, microfluidized formulations maintained relatively stable zeta potentials and exhibited higher encapsulation efficiencies than membrane-extruded liposomes, supporting their superior structural integrity. These improvements can be attributed to the intense shear, cavitation and impact forces generated during microfluidization, which promote more efficient disruption and reassembly of phospholipid bilayers, thereby facilitating the formation of uniformly dispersed nanovesicles with improved peptide entrapment [18]. Based on the combined evaluation of particle size, zeta potential and encapsulation efficiency, high-pressure microfluidization was adopted for subsequent formulation development.

Following selection of the preparation strategy, different lipid matrixes composed of egg yolk lecithin or soybean phospholipids combined with cholesterol or stigmasterol were comparatively evaluated (Table 1). Although all formulations exhibited negative surface charges, noticeable differences were observed in particle size and peptide encapsulation. Compared with cholesterol-containing formulations, the incorporation of stigmasterol generally produced smaller nanoliposomes while maintaining comparable or improved encapsulation efficiencies. This is likely associated with its distinct sterol structure, which may modulate phospholipid organization and membrane properties, thereby contributing to improved peptide retention during vesicle formation [19]. Rather than serving as a simple replacement for cholesterol, stigmasterol provides an alternative approach for modulating membrane properties and improving the retention of highly water-soluble collagen peptides within phospholipid vesicles.

The influence of stigmasterol proportion on nanoliposome formation was further investigated. Increasing the stigmasterol content from 0 to 3% markedly reduced particle size while simultaneously enhancing encapsulation efficiency, suggesting that moderate incorporation of stigmasterol improved vesicle formation and peptide retention, possibly through regulation of lipid organization within the membrane structure [20]. In contrast, zeta potential remained nearly unchanged over the investigated sterol range, indicating that the improved encapsulation performance was unlikely to be associated with changes in surface charge and may be related to alterations in membrane structure and organization. Further increases in stigmasterol content produced only marginal changes in particle size and encapsulation efficiency, implying that the membrane-condensing effect of stigmasterol gradually approached saturation. A similar trend was observed for encapsulation efficiency. In the absence of stigmasterol, encapsulation efficiency remained below 10%, highlighting the limited ability of phospholipid-only membranes to retain highly water-soluble peptides. Increasing the sterol proportion to 3% significantly enhanced encapsulation efficiency to approximately 22%, after which no further improvement was observed. The concurrent decrease in particle size and increase in encapsulation efficiency suggest that stigmasterol improved membrane organization and reduced peptide leakage from the liposomal interior. This observation is consistent with previous reports describing the ability of sterols to regulate phospholipid organization and membrane properties [21]. Therefore, a stigmasterol proportion of 3% (*w*/*w* of total lipids) was adopted for subsequent formulation development.

### 2.2. Establishment of Formulation Composition and Processing Conditions

Following the selection of the lipid matrix, the formulation composition was further established by varying the initial CP concentration and phospholipid dosage. As shown in Table 2, increasing the phospholipid dosage consistently enhanced encapsulation efficiency at all initial CP concentrations, indicating that sufficient phospholipid availability facilitated the formation of complete lipid bilayers capable of retaining hydrophilic peptides. More importantly, increasing the initial CP concentration markedly improved the absolute peptide loading of the nanoliposomes. Although formulations prepared with lower peptide concentrations generally exhibited satisfactory encapsulation efficiencies, their actual peptide payload remained limited because of the relatively low amount of peptide introduced into the system. In contrast, the 40% (*w*/*v*) CP formulation achieved substantially greater peptide loading while maintaining favorable encapsulation efficiency, demonstrating that highly concentrated collagen peptide solutions remained compatible with liposome self-assembly. This characteristic is particularly advantageous for practical production, as collagen peptide concentrates generated during industrial processing are commonly handled at similarly high CP contents [22].

Changes in particle size and zeta potential further supported the selection of the formulation composition. Increasing phospholipid dosage progressively reduced particle size and generated more negative zeta potentials, indicating the formation of more compact lipid bilayers with enhanced colloidal stability. Even at the highest peptide concentration investigated, the nanoliposomes maintained nanoscale particle sizes and sufficiently high absolute zeta potentials, suggesting that increasing peptide loading did not compromise structural stability. Considering peptide loading capacity together with physicochemical stability, an initial CP concentration of 40% (*w*/*v*) and a phospholipid-to-peptide mass ratio of 1:2 was adopted for subsequent preparation.

The influence of microfluidization pressure on the selected formulation was subsequently investigated. Increasing homogenization pressure from 40 to 80 MPa effectively reduced particle size, whereas further increases resulted in slightly larger particles with only marginal improvements in encapsulation efficiency. This phenomenon is consistent with the over-processing behavior frequently observed in high-energy homogenization systems, where excessive mechanical energy promotes vesicle recoalescence during membrane reassembly [23]. In contrast, zeta potential remained relatively stable throughout the investigated pressure range, indicating that increasing mechanical energy had little influence on the surface characteristics of the nanoliposomes. Therefore, a homogenization pressure of 80 MPa was adopted for subsequent preparation. The effect of microfluidization cycles exhibited a similar trend. Increasing the number of homogenization cycles progressively reduced particle size, reflecting more complete disruption of residual large vesicles and improved particle uniformity. Meanwhile, encapsulation efficiency increased initially and reached a maximum after three cycles before gradually declining upon further processing. These results suggest that moderate repeated homogenization promotes membrane reorganization and peptide entrapment, whereas excessive processing destabilizes previously formed vesicles and facilitates peptide leakage [24]. As observed for homogenization pressure, zeta potential remained essentially unchanged throughout the investigated cycle range. Accordingly, 3 homogenization cycles were adopted for all subsequent experiments.

Collectively, the established formulation demonstrated that an appropriate balance among peptide concentration, peptide retention, and colloidal stability could be achieved through rational adjustment of formulation composition and microfluidization conditions. Considering the highly water-soluble nature of collagen peptides, increasing the initial peptide concentration is critical for improving the peptide content of the final nanoliposomal dispersion, although further enhancement of peptide-to-lipid utilization efficiency remains an important consideration for future scale-up applications. The optimized nanoliposomes, prepared using 40% (*w*/*v*) CP, a phospholipid-to-peptide mass ratio of 1:2, a homogenization pressure of 80 MPa, and three microfluidization cycles, were subsequently employed for structural characterization, gastrointestinal digestion, and in vivo bioavailability studies.

### 2.3. Purification, Spray Drying, and Physicochemical Characterization of CP-Loaded Nanoliposomes

**Figure 1:**
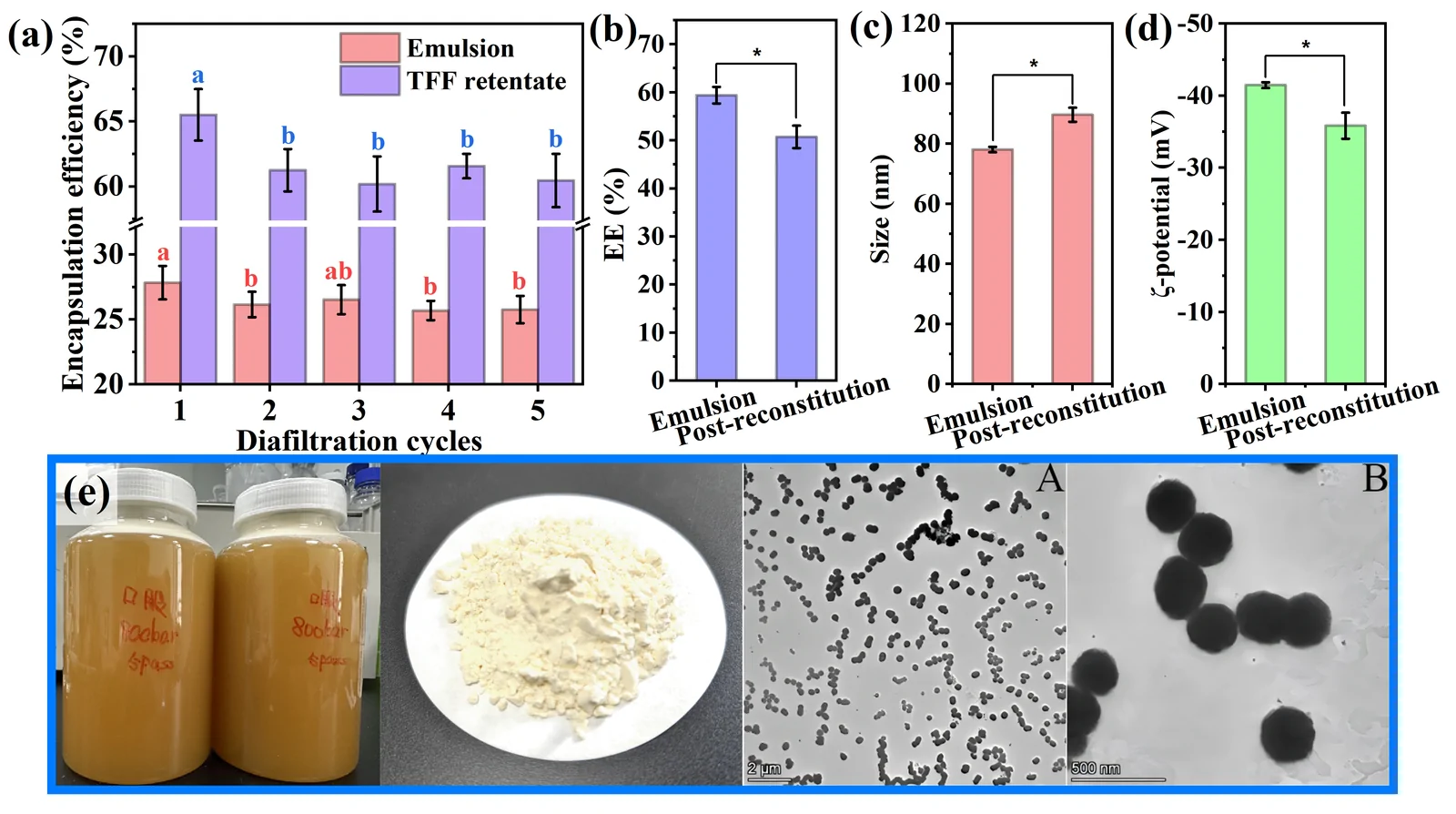
Purification, spray-drying, and morphological characterization of optimized NCP. (**a**) Apparent encapsulation efficiency of crude emulsion and purified nanoliposomes across 5 TFF diafiltration cycles. Comparison of (**b**) encapsulation efficiency, (**c**) particle size, and (**d**) zeta potential between initial liquid emulsions and spray-dried/reconstituted formulations. (**e**) Visual appearance of liquid and reconstituted dispersions, morphology of spray-dried powder, and transmission electron microscopy (TEM) micrographs of reconstituted vesicular nanoliposomes, (**A**) Low-magnification TEM image and (**B**) high-magnification TEM image of the reconstituted NCP. Data are presented as mean ± standard deviation (*n* = 3). Different lowercase letters and * indicate significant differences determined by one-way ANOVA followed by LSD post hoc test at *p* < 0.05.

Although the optimized microfluidization process enabled the successful preparation of CP-loaded nanoliposomes, a substantial proportion of CPs remained unencapsulated because of their high hydrophilicity. Consequently, free peptides and other low-molecular-weight components coexisted with liposome-associated peptides in the crude dispersion. To enrich the nanoliposome fraction and improve product purity, TFF was introduced as a post-processing purification step [25]. As shown in Figure 1a, TFF treatment markedly increased the apparent encapsulation efficiency of the nanoliposome system. This increase should be attributed to the removal of unencapsulated peptides and enrichment of the liposome-associated peptide fraction during purification, rather than enhanced peptide incorporation into the lipid bilayer. Following a single filtration cycle, the apparent encapsulation efficiency increased from approximately 28% to more than 65%, reflecting the efficient removal of non-encapsulated peptides from the continuous phase. Additional filtration cycles produced no significant improvement, with values remaining within the range of 60–65%. A slight decline after repeated filtration may reflect partial peptide leakage resulting from prolonged circulation through the filtration system. Therefore, a single TFF cycle was considered sufficient to achieve effective purification while minimizing processing time and energy consumption.

To facilitate long-term storage and practical application, the purified CP-loaded nanoliposome dispersion was subsequently converted into a dry powder by spray drying. The physicochemical properties of the reconstituted powder were compared with those of freshly prepared nanoliposomes to evaluate the structural stability of the system during dehydration and rehydration. As illustrated in Figure 1b–d, spray drying induced only minor changes in the physicochemical characteristics of the NCP. After reconstitution, the measured encapsulation efficiency decreased slightly but remained at a relatively high level (Figure 1b), indicating that most liposome-associated peptides were retained during the drying and rehydration processes. Similarly, the average particle size increased modestly while remaining within the nanoscale range (Figure 1c). Such changes are commonly observed in spray-dried liposomal systems and are generally attributed to limited vesicle fusion or structural rearrangement occurring during dehydration and subsequent rehydration [26]. Meanwhile, the absolute value of the zeta potential decreased slightly after reconstitution (Figure 1d). Nevertheless, all formulations maintained an absolute ζ-potential above 30 mV, suggesting that sufficient electrostatic repulsion was preserved to maintain colloidal stability.

To further characterize the molecular interactions within the nanoliposomal system and evaluate the thermal behavior of the spray-dried powder, FT-IR and TG/DSC analyses were performed. As shown in Figure S1, collagen peptides exhibited characteristic absorption bands corresponding to amide I (~1650 cm^−1^), and amide II (~1540 cm^−1^), while blank nanoliposomes showed typical lipid-related signals, including C–H stretching vibrations (2924 and 2853 cm^−1^), C=O stretching (~1740 cm^−1^), and P=O stretching (~1240 cm^−1)^. The FT-IR spectrum of NCP retained the characteristic signals of both collagen peptides and phospholipid components, confirming the successful incorporation of CP into the nanoliposomal system. Moreover, the broadening and slight shift in the hydroxyl/amide-related absorption region suggested the involvement of non-covalent interactions between peptides and lipid components. No new characteristic peaks associated with chemical bond formation were observed, indicating that peptide incorporation occurred mainly through physical interactions.

The thermal behavior of spray-dried NCP powder was further evaluated by TG/DSC analysis (Figure S2). A minor weight loss before 150 °C was observed, which was mainly attributed to the removal of residual moisture. The major degradation stage occurred between 200 and 500 °C, accompanied by the maximum degradation rate at approximately 335 °C in the DTG curve, corresponding to the thermal decomposition of organic components. The absence of abnormal thermal transitions within the processing-related temperature range suggested that spray drying generated a relatively stable powder formulation and preserved the physicochemical characteristics of the nanoliposomal system.

The morphological characteristics of the reconstituted CP-loaded nanoliposomes were further examined by transmission electron microscopy (TEM). As shown in Figure 1e, the particles were uniformly dispersed without obvious aggregation and exhibited well-defined spherical or near-spherical vesicular structures. Distinct membrane boundaries were clearly visible, indicating that the liposomal architecture remained largely intact following spray drying and reconstitution. These observations were consistent with the minimal changes observed in encapsulation efficiency, particle size, and zeta potential, supporting the structural stability of the nanoliposomes during dehydration and rehydration [27].

TFF effectively enriched the liposome-associated peptide fraction through purification, whereas spray drying enabled the conversion of the purified dispersion into a stable powder form with only minor alterations in physicochemical properties. Together with the preserved vesicular morphology observed by TEM, these results demonstrate that CP-loaded nanoliposomes can be processed into a dry formulation while maintaining their essential structural and colloidal characteristics. Therefore, the purified and spray-dried nanoliposomes were selected for subsequent evaluation of gastrointestinal stability and oral bioavailability.

### 2.4. Stability of Reconstituted NCP Under Storage and Processing Conditions

**Figure 2:**
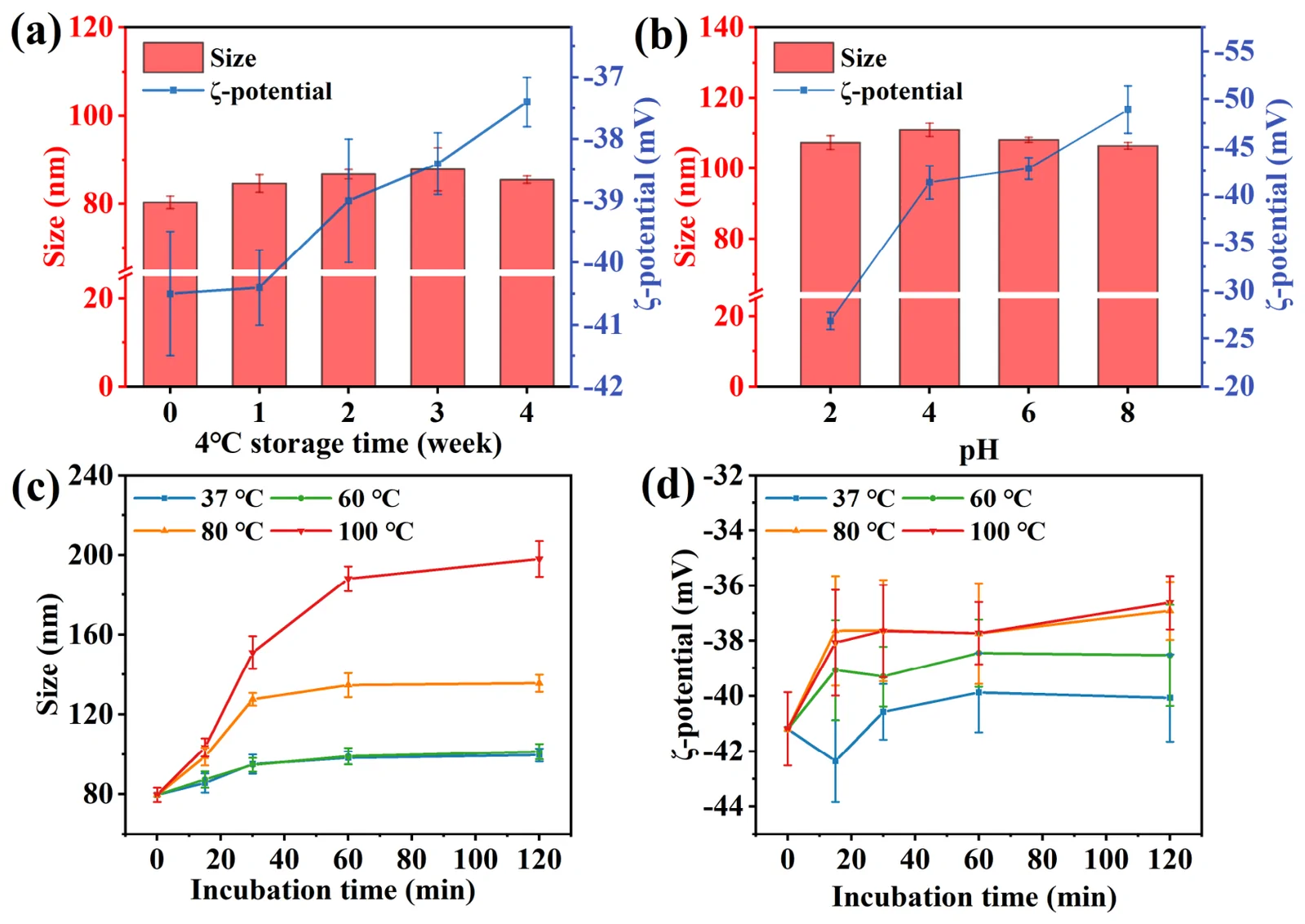
Physicochemical stability profiles of reconstituted NCP under various storage and environmental conditions. Changes in particle size and zeta potential during (**a**) 4-week refrigerated storage at 4 °C, (**b**) incubation across a broad pH range of 2.0 to 8.0, and thermal treatment at varying temperatures (37, 60, 80, and 100 °C) regarding (**c**) particle size growth and (**d**) zeta potential alterations over 120 min. Data are presented as mean ± standard deviation (*n* = 3).

The practical application of nanoliposome delivery systems largely depends on their ability to maintain structural integrity during storage and food processing. Therefore, the stability of reconstituted NCP was systematically evaluated under refrigerated storage, different pH environments, and thermal treatments (Figure 2). As shown in Figure 2a, NCP exhibited excellent storage stability under refrigerated conditions (4 °C). During four weeks of storage, the average particle size remained within 80–90 nm, showing only minor fluctuations, while the ζ-potential decreased slightly from −40.5 mV to −37.5 mV. Despite this small reduction, the absolute ζ-potential remained well above the generally accepted colloidal stability threshold (30 mV), indicating that sufficient electrostatic repulsion was maintained throughout storage [28]. The negligible changes in particle size suggest that the phospholipid bilayer remained structurally intact and effectively prevented particle aggregation or fusion at low temperature.

To appreciate its processing resilience in practical applications, the pH stability of NCP was evaluated over a wide range of pH 2.0–8.0, a representative spectrum mimicking most commercial food and beverage matrices (Figure 2b). Notably, the particle size remained highly stable, fluctuating only between 105 and 110 nm, demonstrating excellent resistance to both acidic and alkaline environments. Such stability is particularly important for oral delivery applications, as it indicates that the nanoliposomes can tolerate the strongly acidic gastric environment without significant aggregation [29]. In contrast, the ζ-potential showed a clear pH-dependent response, increasing in magnitude from −26.90 mV at pH 2.0 to −48.92 mV at pH 8.0. This behavior can be attributed to the protonation and deprotonation of ionizable groups on the phospholipid surface [30]. Although the electrostatic repulsion was partially weakened under strongly acidic conditions, no significant increase in particle size was observed, suggesting that steric stabilization provided by the phospholipid–stigmasterol bilayer also contributed substantially to maintaining colloidal stability.

Thermal stability was assessed by monitoring particle size and ζ-potential during incubation at 37, 60, 80, and 100 °C (Figure 2c,d). At 37 and 60 °C, NCP remained highly stable throughout the 120 min treatment period, with only negligible changes in particle size and surface charge. These results indicate that the nanoliposomes can readily withstand physiological temperature and conventional pasteurization conditions [31]. However, when the temperature increased to 80 °C and 100 °C, a pronounced increase in particle size was observed. At 80 °C, particle size rapidly increased to 135.52 ± 0.17 nm before reaching a plateau, whereas treatment at 100 °C resulted in continuous growth to nearly 200 nm. Concurrently, the absolute ζ-potential decreased to −36.80 ± 1.13 mV. The observed thermal destabilization is likely associated with increased membrane fluidity and partial phase transition of the phospholipid bilayer, which promotes vesicle fusion and particle enlargement. Nevertheless, even after exposure to 100 °C for 120 min, the ζ-potential remained above 30 mV in magnitude, and no severe precipitation or complete structural collapse was observed. NCP demonstrated excellent refrigerated storage stability, broad pH tolerance, and satisfactory thermal resistance. The system remained structurally stable under conditions relevant to refrigerated storage, gastrointestinal transit, and conventional food processing, highlighting its potential as a practical oral delivery platform for CPs.

### 2.5. Colloidal Behavior Changes of Nanoliposomes During Simulated Gastrointestinal Digestion

**Figure 3:**
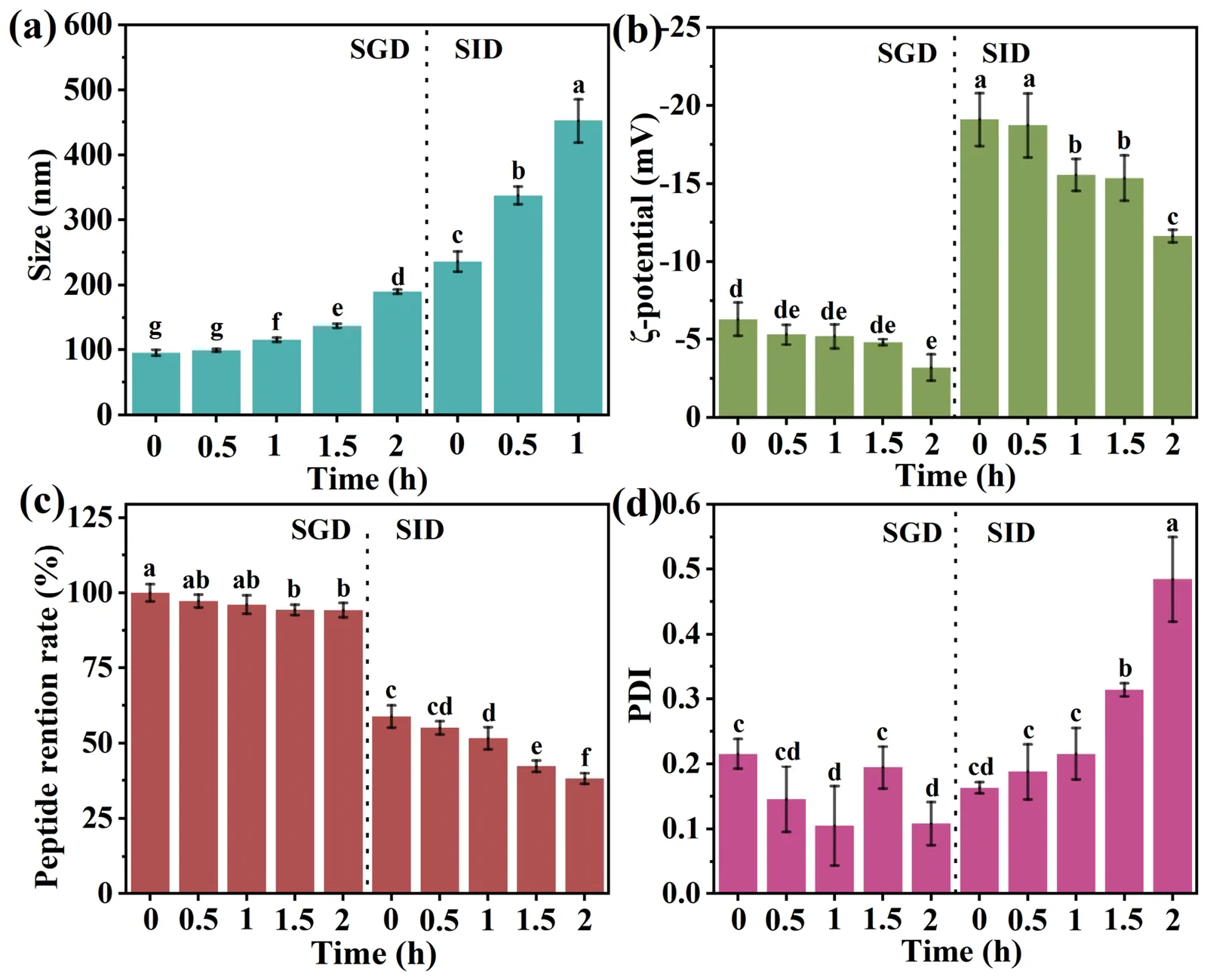
Dynamic colloidal behavior changes of NCP during simulated static in vitro gastrointestinal digestion. Evolution of (**a**) particle size, (**b**) zeta potential, (**c**) peptide retention rate, and (**d**) polydispersity index during simulated gastric digestion (SGD, 0–2 h) and simulated intestinal digestion (SID, 0–2 h). Data are presented as mean ± standard deviation (*n* = 3). Different lowercase letters indicate significant differences determined by one-way ANOVA followed by LSD post hoc test at *p* < 0.05.

To evaluate the stability and release behavior of NCP, their colloidal properties were monitored throughout simulated gastric digestion (SGD) and simulated intestinal digestion (SID). During the gastric phase (0–2 h), NCP remained relatively stable. The particle size gradually increased from 95.37 ± 4.56 nm to 189.80 ± 3.27 nm (Figure 3a), while the PDI remained below 0.2 throughout digestion (Figure 3d), indicating limited aggregation and maintenance of a relatively homogeneous particle population. Meanwhile, the ζ-potential remained between −3 and −6 mV (Figure 3b), which may be attributed to protonation of ionizable phospholipid head groups under acidic conditions, resulting in partial charge neutralization and reduced electrostatic repulsion [32]. The peptide retention rate showed a slight decrease of approximately 5.74% (Figure 3c), demonstrating that most CPs remained associated with the nanoliposomes during gastric digestion.

**Figure 4:**
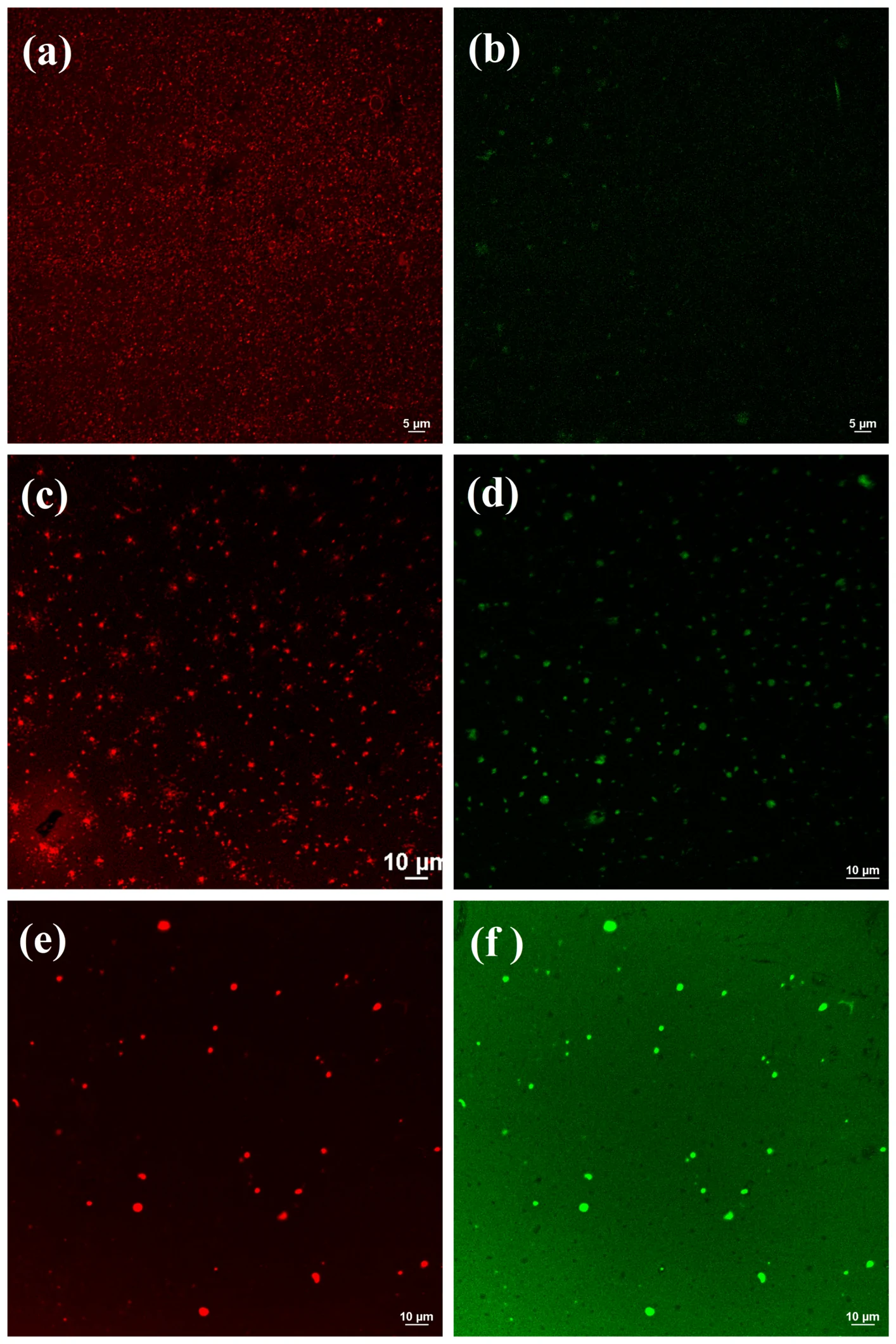
CLSM images showing the structural evolution of NCP. (**a**,**b**) Before digestion; (**c**,**d**) after 2 h SGF; (**e**,**f**) after 2 h SIF. The left column shows phospholipid membranes stained with Nile Red, and the right column shows CPs stained with Nile Blue–NaOH. The white scale bars represent 5 μm in (**a**,**b**) and 10 μm in (**c**–**f**).

The CLSM observations further confirmed the structural stability of NCP during gastric digestion (Figure 4). Before digestion, Nile Red staining revealed uniformly dispersed lipid vesicles with relatively homogeneous fluorescence, while CPs stained by Nile Blue were predominantly confined within discrete fluorescent domains that largely overlapped with the lipid phase, indicating successful peptide encapsulation within the nanoliposomes. Following 2 h of gastric digestion, both lipid membrane and peptide fluorescence remained clearly visible. Although the fluorescent particles became slightly larger and more dispersed, their overall spherical morphology was preserved, consistent with the moderate increase in particle size determined by dynamic light scattering. Moreover, the peptide fluorescence remained highly colocalized with the lipid phase, suggesting that the phospholipid–stigmasterol membrane effectively retained CPs under acidic gastric conditions and minimized premature peptide leakage.

Upon transfer to the intestinal phase, pronounced structural changes were observed. The particle size rapidly increased to 452.30 ± 33.27 nm, accompanied by a substantial increase in PDI to 0.485, indicating loss of monodispersity and significant particle restructuring. Concurrently, the ζ-potential initially decreased to −19.10 ± 1.70 mV before gradually increasing to −11.63 ± 0.40 mV (Figure 3b), reflecting dynamic interactions between the nanoliposomes and intestinal digestion components. Correspondingly, the peptide retention rate declined sharply from 94.26 ± 2.40% to 58.90 ± 3.68% during the gastric-to-intestinal transition and further decreased to 38.19 ± 1.76% after 2 h of intestinal digestion (Figure 3c).

These physicochemical changes were directly reflected in the CLSM images (Figure 4). Compared with the gastric stage, the number of intact lipid vesicles decreased markedly after intestinal digestion, while the remaining fluorescent particles became substantially larger and more irregular in morphology. At the same time, peptide fluorescence became more diffusely distributed throughout the observation field and exhibited reduced colocalization with the lipid membrane, indicating progressive dissociation of CPs from the nanoliposomes. Rather than undergoing immediate disintegration, the liposomal vesicles appeared to experience gradual swelling, fusion and structural remodeling before extensive peptide release occurred. This structural evolution agrees well with the increase in particle size, loss of colloidal homogeneity and reduction in peptide retention rate observed during intestinal digestion.

The observed behavior can be attributed to the combined actions of bile salts and pancreatic enzymes. Bile salts are readily inserted into phospholipid bilayers and promote the formation of mixed micelles, whereas pancreatic lipases hydrolyze membrane phospholipids into lysophospholipids and free fatty acids, collectively destabilizing the lipid membrane. Consequently, the nanoliposomes undergo progressive structural reorganization, ultimately facilitating the release of encapsulated CPs [33]. Similar behavior has been widely reported for phospholipid-based delivery systems undergoing intestinal digestion. Collectively, these results indicate that NCP improved peptide retention during gastric digestion while enabling substantial peptide release under intestinal conditions, which is favorable for peptide absorption in the small intestine.

### 2.6. Plasma Pharmacokinetics of Characteristic Hydroxyproline-Containing Peptides

Previous studies have demonstrated the potential of nanoliposomes for improving the delivery performance of bioactive peptides; however, these systems differ considerably in terms of lipid composition, peptide properties, and evaluation endpoints. For example, sodium alginate-coated collagen peptide liposomes have been reported to improve gastrointestinal stability and in vitro intestinal permeability, whereas cod-derived osteogenic peptide nanoliposomes enhanced peptide bioaccessibility and preserved biological functionality after encapsulation [10,11]. More recently, wheat peptide nanoliposomes prepared using phosphatidylethanolamine and cholesterol achieved high encapsulation efficiency and improved stability under simulated gastrointestinal conditions [12]. Nevertheless, most previous studies have primarily focused on formulation characteristics, physicochemical stability, digestion behavior, or in vitro bioactivity, while the systemic fate and molecular forms of circulating peptides following oral administration remain insufficiently characterized.

**Figure 5:**
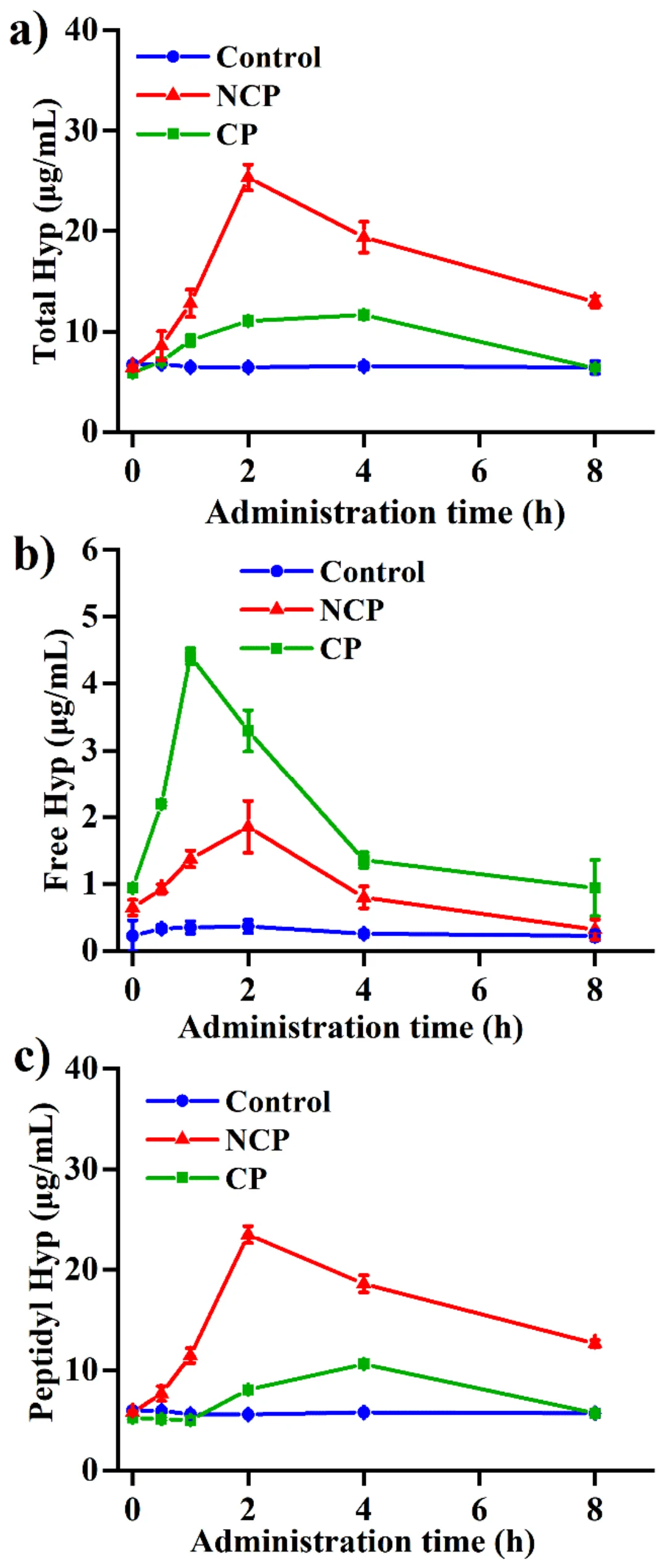
In vivo plasma pharmacokinetics and systemic absorption forms of Hyp following oral administration. Plasma concentration–time profiles of (**a**) total hydroxyproline, (**b**) free hydroxyproline, and (**c**) peptide-bound hydroxyproline in rats after a single gavage of physiological saline (Control), unencapsulated collagen peptides (CP), and collagen peptide-loaded nanoliposomes (NCP). Data are presented as mean ± standard deviation (*n* = 6).

Hydroxyproline is rarely found in conventional dietary proteins and therefore serves as a specific biomarker for tracing the absorption and metabolic fate of orally administered CPs. In circulation, absorbed hydroxyproline exists as both free hydroxyproline and peptide-bound hydroxyproline, the latter mainly comprising hydroxyproline-containing dipeptides and tripeptides that are considered key bioactive forms responsible for the physiological functions of CPs [34]. To evaluate the influence of nanoliposome delivery on the in vivo absorption behavior of CPs, plasma concentration–time profiles of total, free, and peptide-bound hydroxyproline were determined following oral administration (Figure 5), and the corresponding pharmacokinetic parameters were calculated (Table 3).

As shown in Figure 5a, the plasma concentration profiles of total hydroxyproline differed markedly between the CP and NCP groups. Free CPs exhibited relatively slow absorption, reaching a maximum plasma concentration at 4 h after administration. In contrast, NCP displayed substantially improved pharmacokinetic characteristics, with the peak concentration occurring at 2 h and reaching a significantly higher level than that of the CP group. Consistent with these observations, the pharmacokinetic analysis revealed that the iAUC_0−8 h_ of the NCP group reached 83.13 h·μg/mL, representing a 3.84-fold increase compared with the CP group (21.63 h·μg/mL). Meanwhile, the Cmax increased from 11.65 to 25.33 μg/mL, whereas the Tmax decreased from 4 h to 2 h. These results demonstrate that nanoliposomal encapsulation substantially enhances both the absorption rate and overall systemic exposure of orally administered CPs.

To further elucidate the absorption form of CPs, plasma hydroxyproline was differentiated into free and peptide-bound fractions. As shown in Figure 5b, oral administration of free CPs resulted in a rapid increase in plasma free hydroxyproline, reaching its maximum concentration within 1 h. This finding suggests that a considerable proportion of unprotected peptides underwent extensive hydrolysis in the gastrointestinal tract before absorption, generating free amino acids that were subsequently transported across the intestinal epithelium. In contrast, the NCP group maintained a remarkably low free hydroxyproline level throughout the experimental period, with an iAUC_0–8 h_ value of only 5.21 h·μg/mL, corresponding to approximately 39% of that observed in the CP group. A markedly different trend was observed for peptide-bound hydroxyproline (Figure 5c). The NCP group exhibited a sharp increase in peptide-bound hydroxyproline concentration, reaching a maximum at 2 h and maintaining significantly higher levels than the CP group throughout the monitoring period. Notably, the iAUC_0−8 h_ of peptide-bound hydroxyproline increased from 8.35 h·μg/mL in the CP group to 77.92 h·μg/mL following nanoliposomal delivery, corresponding to a 9.3-fold enhancement. Furthermore, peptide-bound hydroxyproline accounted for approximately 94% of the total absorbed hydroxyproline in the NCP group, indicating that a larger proportion of collagen-derived components remained in peptide-associated forms rather than being completely degraded into free amino acids before systemic exposure.

The predominance of peptide-bound hydroxyproline in the NCP group is consistent with the in vitro gastrointestinal digestion results presented in Figure 3. During digestion, the phospholipid bilayer effectively protected encapsulated CPs from extensive enzymatic hydrolysis, thereby preserving a larger proportion of oligopeptides during gastrointestinal transit [35]. As a consequence, more intact peptide fragments remained available for absorption in the small intestine, resulting in the markedly elevated plasma peptide-bound hydroxyproline levels observed in vivo. Beyond enzymatic protection, the altered distribution between free and peptide-bound hydroxyproline also suggests that nanoliposomal delivery may modify the intestinal transport pathway of CPs. Another notable feature of the NCP group was the prolonged persistence of peptide-bound hydroxyproline in circulation. While hydroxyproline concentrations in the CP group approached baseline levels after 8 h, substantial levels remained detectable in the NCP group. This prolonged systemic exposure indicates that nanoliposomal delivery not only accelerates peptide absorption but also extends the residence time of bioactive collagen-derived peptides in vivo. Such sustained exposure may increase the likelihood of peptide accumulation in peripheral target tissues and contribute to enhanced physiological efficacy following oral administration [36].

**Figure 6:**
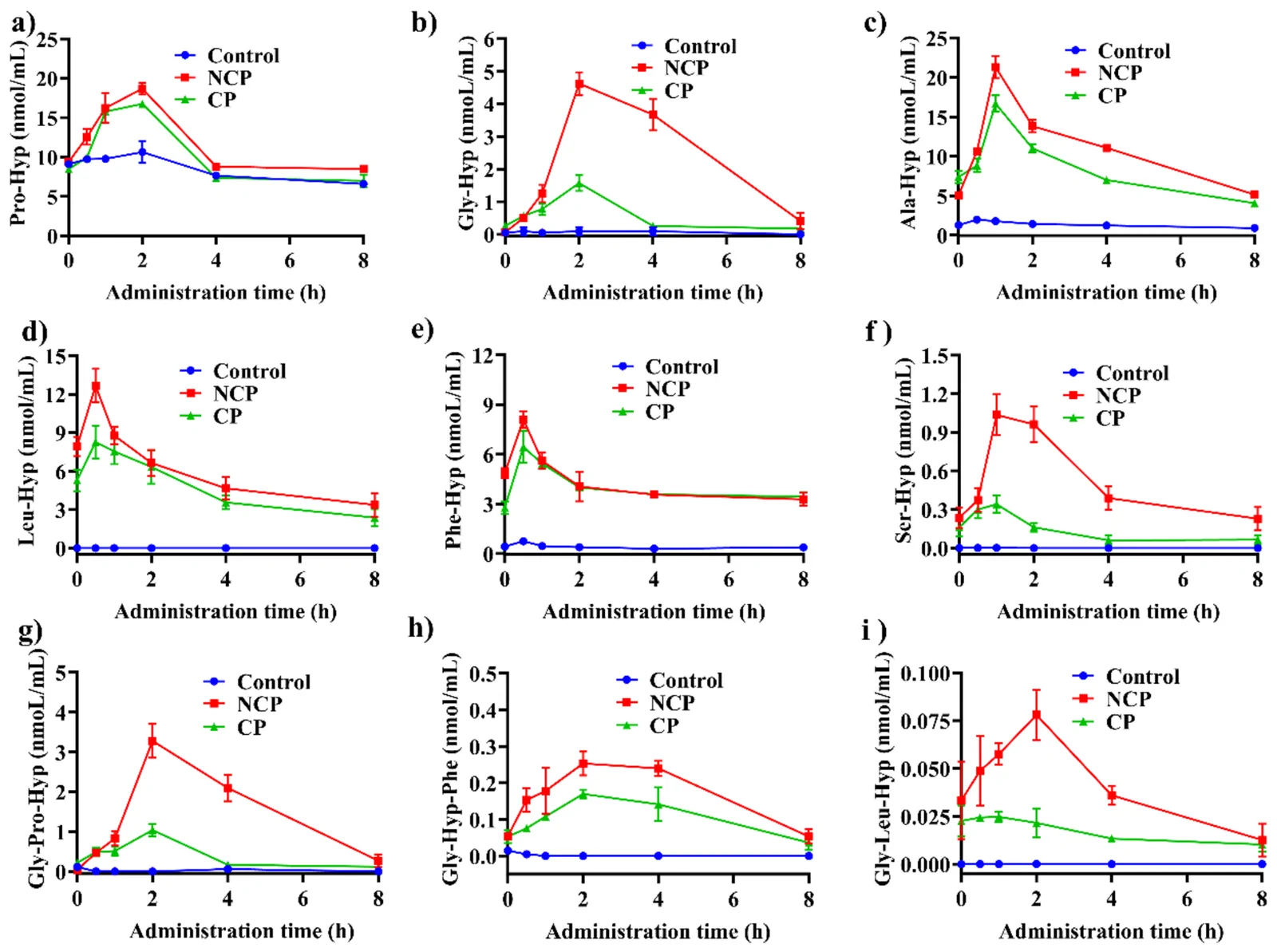
Plasma concentration–time profiles of typical hydroxyproline (Hyp)-containing dipeptides and tripeptides, including (**a**) Pro-Hyp, (**b**) Gly-Hyp, (**c**) Ala-Hyp, (**d**) Leu-Hyp, (**e**) Phe-Hyp, (**f**) Ser-Hyp, (**g**) Gly-Pro-Hyp, (**h**) Gly-Hyp-Phe, and (**i**) Gly-Hyp-Leu, in rats after a single oral administration of physiological saline (Control), unencapsulated collagen peptides (CP), and collagen peptide-loaded nanoliposomes (NCP). Data are presented as mean ± standard deviation (*n* = 6).

To further characterize whether nanoliposomal delivery promoted the systemic absorption of intact collagen-derived peptides, plasma concentrations of six characteristic hydroxyproline-containing dipeptides and three tripeptides were quantified by UHPLC–HRMS following oral administration (Figure 6). Compared with free CPs, NCP markedly increased the plasma exposure of most characteristic peptides, demonstrating that nanoliposomal encapsulation not only enhanced overall peptide absorption but also promoted the systemic availability of specific bioactive peptide species.

Among the detected dipeptides, Pro–Hyp, Ala–Hyp, Leu–Hyp, Phe–Hyp, Gly–Hyp, and Ser–Hyp all exhibited higher plasma concentrations following nanoliposomal delivery (Figure 6a–f). The enhancement was particularly evident for Gly–Hyp and Ser–Hyp, whose peak concentrations increased several-fold compared with those of the CP group. In addition to the increased peak concentrations, several dipeptides, including Pro–Hyp and Gly–Hyp, displayed delayed peak times and broader concentration–time profiles after encapsulation, indicating prolonged absorption and sustained systemic exposure [37]. These findings suggest that nanoliposomal encapsulation not only improves peptide survival during gastrointestinal transit but also modulates their absorption kinetics.

More pronounced differences were observed for hydroxyproline-containing tripeptides (Figure 6g–i). Gly–Pro–Hyp, one of the most extensively studied bioactive collagen-derived tripeptides, showed only minimal plasma exposure following administration of free CPs, whereas nanoliposomal delivery resulted in a dramatic increase in both peak concentration and overall exposure. Similar trends were observed for Gly–Hyp–Phe and Gly–Hyp–Leu, indicating that the phospholipid–stigmasterol bilayer effectively protected these readily hydrolyzed tripeptides during gastrointestinal digestion and facilitated their absorption into the systemic circulation. Because tripeptides are generally more susceptible than dipeptides to enzymatic degradation at the brush-border membrane, the substantially greater enhancement observed for tripeptides further supports the protective role of nanoliposomal encapsulation against gastrointestinal digestion [38].

Notably, the enhancement varied among peptide species. Pro–Hyp exhibited only a moderate improvement following encapsulation, which is consistent with its well-documented intrinsic resistance to enzymatic hydrolysis and relatively high oral bioavailability even in the free form. In contrast, peptides with low plasma exposure in the CP group generally showed much greater enhancement after nanoliposomal delivery, indicating that the delivery system primarily benefited peptide species that are more vulnerable to gastrointestinal degradation [39]. These peptide-specific pharmacokinetic profiles complement the hydroxyproline analysis shown in Figure 5. Whereas plasma hydroxyproline measurements demonstrated that nanoliposomal delivery increased the overall proportion of peptide-bound hydroxyproline, the UHPLC–HRMS results provide direct molecular evidence that characteristic collagen-derived oligopeptides, particularly bioactive tripeptides, were absorbed into the bloodstream in substantially greater amounts following nanoliposomal encapsulation. Together, these findings indicate that the improved systemic exposure of CPs following nanoliposomal delivery is associated with enhanced preservation and circulation of hydroxyproline-containing peptide species rather than simply increased appearance of free hydroxyproline.

## 3. Materials and Methods

### 3.1. Materials

Collagen peptides derived from tilapia scales, with a protein content (dry basis) exceeding 97% and an average molecular weight of 500–600 Da, were kindly provided by Hainan Huayan Collagen Technology Co., Ltd., (Haikou, China). Soybean phospholipids and stigmasterol were purchased from Macklin Biochemical Co., Ltd., (Shanghai, China), while bovine serum albumin (BSA) standard, Folin–Ciocalteu reagent, and phosphotungstic acid staining solution (for the Lowry assay) were obtained from Beijing Solarbio Science & Technology Co., Ltd., (Beijing, China). Triton X-100 was purchased from Yeasen Biotechnology Co., Ltd., (Shanghai, China). LC-MS-grade acetonitrile, formic acid, and methanol were purchased from Merck Chemicals Co., Ltd. (Shanghai, China). All other chemicals and reagents of analytical grade were purchased from Sinopharm Chemical Reagent Co., Ltd. Ultrapure water (18.2 MΩ·cm) was freshly prepared using a Milli-Q purification system (MilliporeSigma, Burlington, MA, USA) throughout the experiments. A laboratory-scale tangential flow filtration (TFF) system equipped with a 50 kDa regenerated cellulose membrane cassette was used for nanoliposome purification (MilliporeSigma, Burlington, MA, USA).

### 3.2. Preparation and Formulation Screening of CP-Loaded Nanoliposomes

CPs were dissolved in ultrapure water to obtain a 10% (*w*/*v*) aqueous solution. Soybean phospholipids were added at phospholipid-to-peptide mass ratios ranging from 1:2 to 1:50 and hydrated under magnetic stirring at room temperature for 30 min to form coarse dispersions. To facilitate preliminary formulation screening, membrane extrusion was employed to compare different lipid matrixes and sterol proportions. After the formulation composition had been established, the selected formulation was subsequently prepared by high-pressure microfluidization for further optimization and characterization. The dispersions were subsequently processed either by membrane extrusion through a 0.22 μm hydrophilic membrane for 3 consecutive passes or by high-pressure microfluidization at 120 MPa for 5 cycles.

To establish a suitable formulation for subsequent investigations, formulation development was carried out in two sequential stages. First, four lipid matrixes composed phospholipids and sterols were comparatively evaluated under identical preparation conditions (CP concentration of 10%, *w*/*v*; CP:phospholipid:sterol = 200:20:1, *w*/*w*/*w*). The particle size, zeta potential and encapsulation efficiency of the resulting liposomal dispersions were measured to compare the emulsification methods for CP encapsulation [40]. After selecting the lipid composition, the formulation was further developed by varying the initial CP concentration (10–40%, *w*/*v*) together with the phospholipid dosage.

The particle size, polydispersity index (PDI), and zeta potential of the prepared nanoliposomes were determined by dynamic light scattering (DLS) using a Zetasizer Nano ZS90 (Malvern, Worcestershire, UK). Samples were diluted with ultrapure water to 0.2–0.5 mg/mL and filtered through a 0.22 μm hydrophilic membrane before measurement. Particle size and PDI were measured at 25 °C with a scattering angle of 90°, while zeta potential was determined using a folded capillary cell based on the Smoluchowski model. All measurements were performed in triplicate.

The encapsulation efficiency of CPs in nanoliposomes was determined using ultrafiltration coupled with the Lowry protein assay [41]. Freshly prepared nanoliposome dispersions were first diluted tenfold with ultrapure water to facilitate ultrafiltration. An equal volume of 5% (*w*/*v*) Triton X-100 solution was then added to completely disrupt the lipid bilayer, and the total peptide concentration in the resulting dispersion was determined and recorded as C_1_. Separately, an aliquot of the diluted nanoliposome dispersion was transferred to a centrifugal ultrafiltration tube (MWCO 10 kDa) and centrifuged at 2000× *g* for 10 min. The filtrate containing non-encapsulated peptides was collected to determine the concentration of free peptides (C_2_). Blank nanoliposomes prepared without CP were analyzed in parallel, and the corresponding background signal was subtracted. The encapsulation efficiency was calculated according to Equation (1):

(1) $$\mathrm{EE}\;\left(\%\right)\;=\;\frac{C_{1}\text{-}10C_{2}}{C_{1}}\times 100\%\;$$

where (C_1_) is the total peptide concentration after liposome disruption, and (C_2_) is the free peptide concentration in the ultrafiltrate.

To further characterize the peptide incorporation relative to the lipid component, the encapsulated peptide-to-phospholipid ratio (P/L, *w*/*w*) was calculated according to Equation (2):

(2) $$P/L(\%)=\frac{C_{cp}\times EE}{C_{pl}}\times 100\%$$

where (C_cp_) is the initial collagen peptide content, (C_pl_) is the phospholipid content, and EE represents the encapsulation efficiency determined by Equation (1).

After establishing the optimal formulation, the effects of microfluidization conditions on the physicochemical properties of CP-loaded nanoliposomes were further investigated. Homogenization pressure varied from 40 to 200 MPa, and the number of microfluidization cycles ranged from 1 to 5. The final preparation conditions were established based on encapsulation efficiency together with particle size and zeta potential.

### 3.3. Purification, Spray Drying, and Reconstitution Characterization of Nanoliposomes

CP-loaded nanoliposome dispersions obtained after microfluidization were subjected to purification by TFF using a 50 kDa molecular weight cut-off membrane module to remove non-encapsulated peptides and other low-molecular-weight species. The TFF process was conducted in 5 consecutive recycle cycles. In each cycle, the filtrate was collected and analyzed in real time for free collagen peptide and phospholipid concentrations. Based on these measurements, corresponding amounts of peptide and lipid materials were accurately replenished to restore the system to its initial feed composition, including a peptide aqueous phase mass fraction of 40% and a collagen peptide:soybean phospholipid:stigmasterol ratio of 60:30:1. The reconstituted dispersion was then reintroduced into the next round of emulsification/microfluidization to maintain process continuity and improve material utilization efficiency. After TFF purification and compositional readjustment, the purified collagen peptide-loaded nanoliposomes (NCP) dispersion was spray-dried using a centrifugal atomization spray dryer to obtain powder. The spray drying process was performed at an inlet temperature of 180 °C, an outlet temperature of 120 °C, and a feed rate of 1.2 L/h. The obtained powder was collected and stored in vacuum-sealed bags at room temperature under dark conditions until further analysis.

The basic physicochemical properties of the spray-dried NCP powder were further characterized, including moisture content, ash content, protein content, whiteness, and bulk density. Moisture and ash contents were determined using standard gravimetric methods, while protein content was determined by the Kjeldahl method. Whiteness was determined using a colorimeter based on the CIE L* a* b* color space, and bulk density was measured by determining the mass-to-volume ratio of the freely settled powder in a graduated cylinder. The dry matter recovery yield of the spray-dried powder was also determined. The corresponding data are presented in Table S2.

Fourier-transform infrared (FT-IR) spectra were recorded using a Nicolet iS10 spectrometer (Thermo Fisher Scientific, Waltham, MA, USA) to analyze the characteristic functional groups and molecular interactions within the nanoliposomal system. The spray-dried nanoliposome powders were mixed with dried KBr at a mass ratio of 1:50 and compressed into pellets. Spectra were collected in the range of 4000–400 cm^−1^.

Thermogravimetric analysis (TGA) and differential scanning calorimetry (DSC) were performed using a STA 449F3 thermal analyzer equipped with a DSC 200PC module (Netzsch, Selb, Germany). Approximately 10 mg of spray-dried nanoliposome powder was sealed in an aluminum crucible and heated from 20 to 600 °C at a rate of 10 °C min^−1^ under a nitrogen atmosphere. The thermal decomposition behavior and heat flow changes in the formulation were recorded.

The morphology of reconstituted nanoliposomes was examined by transmission electron microscopy (Talos F200X G2, Thermo Fisher Scientific, USA). Samples were diluted with ultrapure water, deposited onto carbon-coated copper grids, and negatively stained with 2% (*w*/*v*) phosphotungstic acid for 60 s. After air-drying at room temperature, the samples were observed under the microscope and micrographs were recorded.

### 3.4. Stability Evaluation of Reconstituted Nanoliposomes

Spray-dried NCP powder was reconstituted in deionized water at a concentration of 5% (*w*/*v*) and vortexed thoroughly to obtain a homogeneous dispersion. The physicochemical stability of the reconstituted nanoliposomes was evaluated under different storage, thermal, and pH conditions. For storage stability evaluation, reconstituted dispersions were transferred into sealed glass vials and stored at either 4 °C or 25 °C for 28 days. Samples were collected on days 0, 7, 14, 21, and 28 for physicochemical characterization. For thermal stability evaluation, freshly prepared dispersions were incubated at 37, 60, 80, or 100 °C for 15, 30, 60, and 120 min, followed by cooling to room temperature prior to analysis. For pH stability evaluation, nanoliposome powder was dispersed in buffer solutions at pH 2.0 (HCl–citrate buffer), pH 4.0 (phosphoric acid–sodium dihydrogen phosphate buffer), and pH 6.0 and 8.0 (citric acid–sodium citrate buffer), followed by equilibration at room temperature for 30 min. Particle size and zeta potential were determined according to the procedure described in Section 2.2. Changes in these parameters were used to evaluate the physicochemical stability of the reconstituted nanoliposomes under different environmental conditions.

### 3.5. In Vitro Gastrointestinal Digestion and Characterization of NCP

The gastrointestinal digestion behavior of TFF-purified NCP was investigated using a standardized static in vitro digestion model based on the INFOGEST protocol [42]. Simulated gastric fluid (SGF) and simulated intestinal fluid (SIF) were prepared according to the reported electrolyte compositions (Table S1). Pepsin and pancreatin were freshly incorporated into SGF and SIF, respectively, together with bile salts and calcium chloride immediately prior to digestion. CP-loaded nanoliposomes were first dispersed in 0.03 mol L^−1^ NaCl solution to obtain a final concentration of 5% (*w*/*v*) and equilibrated at 37 °C for 10 min. Gastric digestion was initiated by mixing the dispersion with an equal volume of preheated SGF, followed by adjustment of the pH to 2.0. The mixture was then incubated at 37 °C under continuous shaking (120 rpm) for 2 h. Aliquots were withdrawn at 30 min intervals throughout the gastric digestion process.

Upon completion of the gastric phase, the pH of the digestion mixture was adjusted to 7.5 using 1.0 mol L^−1^ NaOH, after which an equal volume of preheated SIF was added to initiate intestinal digestion. The samples were further incubated at 37 °C and 120 rpm for an additional 2 h, and aliquots were collected every 30 min. Following digestion, the samples were filtered through a 0.22 μm membrane and subjected to subsequent physicochemical and microstructural analyses.

The evolution of nanoliposome properties during gastrointestinal digestion was monitored by determining particle size, polydispersity index (PDI), zeta potential, and collagen peptide retention rate. The peptide retention rate, representing the percentage of CPs remaining associated with the nanoliposomal fraction relative to that before digestion (defined as 100%), was calculated according to Equation (3):

(3) $$Peptide\;retention\;rate\left(\%\right)=\;\frac{C_{t}}{C_{0}}\times 100\%$$

where (C_t_) is the concentration of CPs retained in the nanoliposomal fraction at the corresponding digestion time point, and (C_0_) is the concentration of CPs retained in the nanoliposomal fraction before digestion.

In addition, the microstructural changes in the nanoliposomes during digestion were visualized using an A1R HD25 confocal laser scanning microscopy (CLSM) (Nikon, Tokyo, Japan). Samples were stained with Nile Red and Nile Blue to label the lipid and aqueous phases, respectively, and incubated in the dark for 10 min before observation. The stained dispersions were mounted on microscope slides and examined using a confocal laser scanning microscope. Fluorescence images were acquired at an excitation wavelength of 543 nm with an emission collection range of 560–620 nm.

### 3.6. In Vivo Bioavailability Evaluation

The oral bioavailability of CPs was evaluated using plasma hydroxyproline as a characteristic marker of collagen-derived peptide absorption. A total of 18 healthy male Sprague–Dawley (SD) rats (8 weeks old, 200 ± 20 g) were obtained and acclimatized for 7 days before the experiment. The rats were maintained under controlled conditions (22 ± 1 °C, relative humidity 50 ± 5%, and a 12 h light/dark cycle) with free access to AIN-93G purified diet and sterile deionized water. After fasting for 12 h (with free access to water), rats were randomly divided into three groups (*n* = 6 per group): saline control (Control), unencapsulated collagen peptides (CP), and collagen peptide-loaded nanoliposomes (NCP). The administration dose was standardized based on hydroxyproline equivalents. Considering the hydroxyproline content of tilapia CPs (8%) and the analytical sensitivity required for plasma determination, the final oral dose was set at 80 mg kg^−1^ body weight (expressed as hydroxyproline equivalents). All formulations were dispersed in sterile physiological saline and administered at a constant gavage volume of 1.0 mL adjusted according to body weight. All animal procedures were approved by the Animal Ethics Committee of Ocean University of China (No. SPXY20250110).

### 3.7. UHPLC–HRMS Analysis of Plasma Samples

Plasma samples were analyzed using an ultra-high-performance liquid chromatography coupled with high-resolution mass spectrometry (UHPLC–HRMS) system to characterize collagen-derived peptides, phospholipid-related compounds, and endogenous metabolites. Chromatographic separation was performed on an ACQUITY UPLC BEH C18 column (100 × 2.1 mm, 1.7 μm) maintained at 40 °C. The mobile phases consisted of acetonitrile (A) and ultrapure water (B). The flow rate was set at 0.4 mL min^−1^ with an injection volume of 2 μL. The gradient elution program was as follows: 20% A (0–3.0 min), 20–50% A (3.0–6.0 min), 50–85% A (6.0–9.5 min), 85–100% A (9.5–14.5 min), followed by 100% A until 17.0 min. A solvent blank was injected after every four plasma samples to minimize carryover and maintain chromatographic stability.

Mass spectrometric detection was performed using an Agilent 6410 triple quadrupole mass spectrometer equipped with a Jet Stream electrospray ionization source (Agilent, Santa Clara, CA, USA). The analysis was conducted in positive ion mode with the following source parameters: sheath gas flow, 10 L min^−1^; sheath gas temperature, 325 °C; nebulizer pressure, 35 psig; drying gas flow, 8 L min^−1^; drying gas temperature, 300 °C; and capillary voltage, 4000 V. Characteristic collagen-derived di- and tripeptides were detected based on their specific precursor-to-product ion transitions. Hydroxyproline-containing dipeptides were detected using *m*/*z* 86.1. Gly-Hyp-Yaa-type tripeptides were identified using *m*/*z* 143 as characteristic product ions, respectively. The precursor ion scanning ranges were set as *m*/*z* 186–298 for Hyp-containing dipeptides, and *m*/*z* 246–352 for Gly-Hyp-Yaa tripeptides, respectively.

Absolute quantification was performed using external calibration curves generated from authentic peptide standards. Method validation was conducted by evaluating linearity, limits of detection (LOD), limits of quantification (LOQ), precision, and accuracy. Calibration curves were established using six concentration levels, and LOD and LOQ were determined based on signal-to-noise ratios of 3 and 10, respectively.

### 3.8. Statistical Analysis

All experiments were conducted with at least three independent replicates unless otherwise specified. Data are presented as mean ± standard deviation (SD). Statistical analyses were performed using SPSS Statistics 22.0 (IBM Corp., Armonk, NY, USA). Prior to one-way analysis of variance (ANOVA), the normality of data distribution and homogeneity of variance were evaluated. Statistical significance was determined using one-way ANOVA followed by Fisher’s least significant difference (LSD) post hoc test at *p* < 0.05. Data visualization and figure preparation were performed using Origin 2021 (OriginLab Corp., Northampton, MA, USA).

## 4. Conclusions

In this study, a stable, cholesterol-free collagen peptide nanoliposome system was successfully developed using soybean phospholipids and stigmasterol through high-pressure microfluidization, followed by tangential flow filtration and spray drying. The optimized formulation exhibited excellent redispersibility and physicochemical stability during storage as well as under a wide range of pH and thermal conditions. During simulated gastrointestinal digestion, the stigmasterol-containing nanoliposomal structure improved peptide retention during the gastric phase and facilitated peptide release under intestinal conditions. Oral administration significantly enhanced the systemic exposure of collagen peptides, increasing plasma exposure of total hydroxyproline by 3.84-fold and peptide-bound hydroxyproline by 9.3-fold compared with free CPs. Moreover, nanoliposomal encapsulation altered the circulating molecular profile of absorbed collagen peptides toward peptide-bound species, which accounted for approximately 94% of total circulating hydroxyproline. These findings demonstrate that stigmasterol-containing nanoliposomes represent a promising approach for improving the oral delivery performance of collagen peptides. Further studies are required to elucidate the specific intestinal transport mechanisms and biological effects associated with this formulation.

## Figures and Tables

**Table 1:** Particle size, ζ-potential, and encapsulation efficiency (EE) of formulations prepared by different emulsification methods, lipid compositions, and sterol proportions. Data are presented as mean ± standard deviation (*n* = 3). Different lowercase letters indicate significant differences determined by one-way ANOVA followed by LSD post hoc test at *p* < 0.05.

Emulsification Method	Phospholipid Peptide Ratio	Size (nm)	ζ-Potential (mV)	EE (%)
Membrane extrusion	1:2	435.37 ± 5.40 ^a^	−17.60 ± 2.52 ^b^	14.48 ± 2.42 ^a^
	1:10	415.83 ± 8.20 ^b^	−18.67 ± 2.15 ^b^	10.44 ± 2.13 ^b^
	1:20	389.53 ± 7.66 ^c^	−19.33 ± 1.80 ^b^	6.50 ± 0.84 ^c^
	1:50	366.67 ± 11.48 ^d^	−0.07 ± 0.37 ^a^	3.72 ± 0.33 ^d^
High-pressure microfluidization	1:2	187.93 ± 4.58 ^c^	−32.93 ± 1.86 ^c^	18.29 ± 0.84 ^a^
	1:10	181.20 ± 7.81 ^c^	−34.03 ± 3.91 ^c^	11.52 ± 1.96 ^b^
	1:20	226.87 ± 5.39 ^b^	−29.50 ± 2.13 ^b^	7.91 ± 1.33 ^c^
	1:50	247.13 ± 6.19 ^a^	−24.30 ± 0.66 ^a^	4.83 ± 1.33 ^d^
Lipid composition				
Egg yolk lecithin	1:10	457.68 ± 7.81 ^a^	−18.73 ± 1.78 ^bc^	11.31 ± 1.51 ^c^
Soybean lecithin		433.97 ± 10.70 ^b^	−15.07 ± 2.06 ^a^	10.67 ± 1.24 ^c^
Egg yolk lecithin + Cholesterol		370.43 ± 5.18 ^d^	−16.34 ± 1.64 ^ab^	19.03 ± 1.54 ^a^
Soybean lecithin + Cholesterol		411.33 ± 10.90 ^c^	−16.92 ± 1.22 ^ab^	18.14 ± 1.82 ^a^
Egg yolk lecithin + Stigmasterol		409.57 ± 4.99 ^c^	−17.32 ± 1.46 ^ab^	17.42 ± 1.32 ^ab^
Soybean lecithin + Stigmasterol		357.07 ± 13.51 ^d^	−19.23 ± 1.41 ^c^	16.21 ± 1.19 ^b^
Sterol proportion in lipids (%)				
0	1:10	513.83 ± 32.52 ^a^	−15.73 ± 1.76 ^a^	8.44 ± 1.45 ^d^
1.5		442.07 ± 37.45 ^b^	−15.70 ± 1.51 ^a^	15.36 ± 2.15 ^c^
3		352.90 ± 31.39 ^d^	−15.97 ± 1.86 ^a^	21.54 ± 1.84 ^ab^
4.5		362.97 ± 15.99 ^cd^	−15.33 ± 1.80 ^a^	23.11 ± 1.74 ^ab^
6		367.17 ± 29.73 ^c^	−15.87 ± 1.26 ^a^	21.05 ± 1.45 ^b^
7.5		388.40 ± 28.35 ^b^	−15.50 ± 1.57 ^a^	20.31 ± 1.45 ^b^
9		396.20 ± 38.71 ^ab^	−16.13 ± 1.78 ^a^	20.74 ± 2.73 ^b^
10.5		410.57 ± 34.23 ^a^	−15.47 ± 2.15 ^a^	21.38 ± 3.54 ^b^
12		412.17 ± 20.81 ^a^	−15.53 ± 1.46 ^a^	23.77 ± 0.89 ^a^

**Table 2:** Optimization of core concentration, lipid-to-peptide ratio, and mechanical homogenization parameters on vesicle properties. Data are presented as mean ± standard deviation (*n* = 3). Different lowercase letters indicate significant differences determined by one-way ANOVA followed by LSD post hoc test at *p* < 0.05.

Collagen Peptide Concentration	Phospholipid Peptide Ratio	Size (nm)	ζ-Potential (mV)	EE (%)
10%	1:5	129.94 ± 6.91 ^a^	−27.58 ± 3.47 ^a^	8.65 ± 2.54 ^e^
	2:5	123.09 ± 6.98 ^ab^	−29.73 ± 1.27 ^ab^	11.46 ± 1.56 ^d^
	3:5	113.18 ± 7.71 ^bc^	−31.84 ± 1.83 ^bc^	14.12 ± 0.88 ^c^
	4:5	101.34 ± 2.73 ^cd^	−33.73 ± 3.62 ^cd^	17.91 ± 1.27 ^ab^
	1:1	102.08 ± 6.55 ^cd^	−34.84 ± 2.65 ^de^	19.27 ± 0.86 ^bc^
	6:5	98.49 ± 7.72 ^d^	−35.41 ± 3.64 ^e^	21.64 ± 1.97 ^a^
	7:5	95.73 ± 4.75 ^d^	−34.65 ± 1.85 ^cd^	20.08 ± 1.47 ^c^
20%	3:10	107.68 ± 5.84 ^a^	−26.83 ± 2.12 ^a^	11.17 ± 1.13 ^c^
	2:5	103.60 ± 4.32 ^ab^	−27.64 ± 2.61 ^a^	12.67 ± 0.98 ^c^
	1:2	96.17 ± 5.56 ^bc^	−31.81 ± 2.88 ^b^	16.79 ± 0.64 ^b^
	3:5	94.08 ± 5.87 ^bc^	−34.31 ± 3.97 ^bc^	17.61 ± 0.92 ^b^
	7:10	90.35 ± 6.74 ^cd^	−35.56 ± 4.22 ^c^	20.64 ± 1.92 ^a^
	4:5	82.48 ± 6.44 ^e^	−35.24 ± 4.10 ^c^	21.15 ± 0.90 ^b^
	9:10	84.97 ± 4.18 ^de^	−33.50 ± 3.30 ^bc^	21.05 ± 1.54 ^b^
30%	3:20	109.98 ± 7.20 ^a^	−23.42 ± 2.22 ^a^	11.65 ± 0.60 ^f^
	1:5	109.56 ± 4.95 ^a^	−26.33 ± 1.42 ^b^	14.90 ± 1.22 ^e^
	1:4	95.00 ± 3.08 ^bc^	−31.56 ± 2.98 ^c^	16.73 ± 1.39 ^de^
	3:10	99.62 ± 2.02 ^ab^	−35.95 ± 2.18 ^d^	20.54 ± 1.77 ^cd^
	7:20	85.25 ± 6.96 ^cd^	−38.77 ± 4.55 ^e^	23.66 ± 1.53 ^b^
	2:5	84.83 ± 4.55 ^cd^	−37.35 ± 4.47 ^de^	26.70 ± 1.31 ^a^
	9:20	81.56 ± 3.13 ^d^	−37.92 ± 3.93 ^de^	25.62 ± 1.19 ^bc^
40%	1:6	105.81 ± 4.21 ^a^	−24.70 ± 1.45 ^a^	13.13 ± 1.47 ^f^
	7:30	101.01 ± 2.32 ^ab^	−30.77 ± 4.02 ^b^	18.42 ± 1.66 ^e^
	3:10	99.79 ± 3.40 ^ab^	−30.10 ± 3.77 ^b^	23.86 ± 2.15 ^d^
	11:30	93.47 ± 7.43 ^bc^	−33.94 ± 2.44 ^bc^	25.91 ± 1.70 ^c^
	13:30	94.86 ± 4.94 ^bc^	−35.57 ± 2.43 ^c^	30.33 ± 1.60 ^b^
	1:2	86.86 ± 4.17 ^cd^	−35.67 ± 3.84 ^c^	33.89 ± 1.21 ^a^
	17:30	79.11 ± 4.38 ^d^	−36.44 ± 1.01 ^c^	33.70 ± 2.23 ^a^
Homogenization pressure				
40	1:2	77.22 ± 1.42 ^c^	−39.63 ± 0.40 ^c^	27.73 ± 0.94 ^b^
80		77.72 ± 0.58 ^c^	−42.07 ± 0.55 ^b^	29.34 ± 0.92 ^ab^
120		86.04 ± 0.94 ^b^	−42.73 ± 0.25 ^ab^	26.14 ± 0.96 ^c^
160		94.84 ± 1.37 ^a^	−43.00 ± 0.26 ^a^	28.48 ± 0.93 ^b^
200		96.32 ± 1.02 ^a^	−42.47 ± 0.35 ^ab^	30.66 ± 0.90 ^a^
Homogenization cycles				
1	1:2	78.08 ± 0.89 ^a^	−41.47 ± 0.40 ^a^	27.71 ± 0.46 ^c^
2		74.23 ± 1.69 ^b^	−41.47 ± 0.90 ^a^	29.07 ± 0.45 ^b^
3		70.86 ± 0.63 ^c^	−41.53 ± 0.59 ^a^	30.37 ± 0.44 ^a^
4		69.71 ± 1.10 ^cd^	−41.07 ± 0.21 ^a^	26.91 ± 0.46 ^c^
5		68.20 ± 0.91 ^d^	−40.77 ± 0.25 ^a^	27.39 ± 0.46 ^c^

**Table 3:** Area under the curve of orally administered CPs in free and nanoliposome forms. Data are presented as mean ± standard deviation (*n* = 6). Asterisks indicate significant differences determined by one-way ANOVA followed by LSD post hoc test at *p* < 0.05.

Group	Hyp Types	iAUC (h·μg/mL)	C_max_ (μg/mL)	T_max_ (h)
CP	Total Hyp	21.6275 ± 1.8076 *	11.6466 ± 0.1595	4
	Free Hyp	13.2791 ± 1.0438	4.4109 ± 0.1171	1
	Pep-bound Hyp	8.3484 ± 0.9316	10.5900 ± 0.1383	4
NCP	Total Hyp	83.1349 ± 6.3701 *	25.3333 ± 1.2799	2
	Free Hyp	5.2132 ± 0.8618	1.8527 ± 0.3873	2
	Pep-bound Hyp	77.9217 ± 6.1433	23.4806 ± 0.8336	2

## Data Availability

The data supporting the findings of this study are available within the article and its Supplementary Materials. Additional data are available from the corresponding author upon reasonable request.

## Supplementary Materials

The following supporting information can be downloaded at https://www.mdpi.com/article/10.3390/md24080293/s1. Figure S1: FT-IR spectra of CP, blank nanoliposomes, and NCP; Figure S2. Thermogravimetric (TG), derivative thermogravimetric (DTG), and differential scanning calorimetry (DSC) curves of NCP; Figure S3. MS/MS spectra of Xaa-Hyp-type di/tripeptides in tilapia scale collagen peptides and NCP; Table S1: Optimization of formulation composition and homogenization parameters on the encapsulated peptide-to-phospholipid ratio (P/L). Table S2. Basic physicochemical properties of spray-dried NCP powder. Table S3. Identification of characteristic di-/tripeptides in tilapia scale collagen peptides and collagen peptide-loaded nanoliposomes (NCP) by UHPLC-HRMS. Table S4. Calibration curves, R2, LOD, and LOQ for hydroxyproline-containing characteristic di- and tripeptides.
