# Supplementary material for: Stigmasterol-Stabilized Nanoliposomes Enhance Oral Delivery of Collagen Peptides Through Improved Systemic Exposure of Hydroxyproline-Containing Peptides
Source: Mar Drugs. 2026 Aug 21;24(8):293. doi: 10.3390/md24080293 (PMC13514345; doi:10.3390/md24080293)
Supplement: Supplementary file 1 [file marinedrugs-24-00293-s001.zip › marinedrugs-4473124-supplementary.pdf]

Table S1. Optimization of formulation composition and homogenization parameters on the encapsulated peptide-to-phospholipid ratio (P/L). Data are presented as mean ± SD (n = 3). Different lowercase letters indicate significant differences determined by one-way ANOVA followed by LSD post hoc test at $p < 0.05$.

Collagen peptide concentration	Phospholipid peptide ratio	P/L (%, w/w)
10%	1:5	43.26 ± 12.72^{a}
	2:5	28.64 ± 3.90^{b}
	3:5	23.54 ± 1.46^{bc}
	4:5	22.39 ± 1.59^{bc}
	1:1	19.27 ± 0.86^{c}
	6:5	18.03 ± 1.64^{c}
	7:5	14.35 ± 1.05^{d}
20%	3:10	37.25 ± 3.77^{a}
	2:5	31.66 ± 2.46^{b}
	1:2	33.58 ± 1.27^{ab}
	3:5	29.36 ± 1.53^{bc}
	7:10	29.49 ± 2.74^{bc}
	4:5	26.44 ± 1.12^{c}
	9:10	23.39 ± 1.71^{d}
30%	3:20	77.69 ± 3.98^{a}
	1:5	74.52 ± 6.12^{ab}
	1:4	66.92 ± 5.57^{b}
	3:10	68.48 ± 5.90^{b}
	7:20	67.59 ± 4.36^{b}
	2:5	66.76 ± 3.27^{b}
	9:20	56.92 ± 2.64^{c}
40%	1:6	78.75 ± 8.83^{a}
	7:30	78.96 ± 7.13^{a}
	3:10	79.52 ± 7.15^{a}
	11:30	70.67 ± 4.65^{b}
	13:30	70.00 ± 3.69^{b}
	1:2	67.78 ± 2.43^{b}
	17:30	59.48 ± 3.93^{c}
Homogenization pressure		
40	1:2	55.45 ± 1.87^{b}
80		58.69 ± 1.83^{ab}
120		52.28 ± 1.91^{c}
160		56.95 ± 1.85^{b}
200		61.33 ± 1.80^{a}
Homogenization cycles		
1	1:2	55.42 ± 0.92^{c}
2		58.13 ± 0.90^{b}
3		60.74 ± 0.88^{a}
4		53.81 ± 0.93^{c}
5		54.79 ± 0.92^{c}

Table S2. Basic physicochemical properties of spray-dried NCP powder.

Parameters	Moisture	Ash content	Protein content	Whiteness	Bulk density	Dry matter recovery yield
	3.76 ± 0.35%	3.72 ± 0.41%	76.41 ± 0.89%	63.7	0.47	71.39%

Table S3. Identification of characteristic di-/tripeptides in tilapia scale collagen peptides and collagen peptide-loaded nanoliposomes (NCP) by UHPLC-HRMS.

Peptide Category	Sequence	Exact Molecular Weight (Da)	Theoretical m/z	Observed m/z	Mass Error (ppm)	Characteristic Fragment Ions
Xaa-Hyp	Gly-Hyp	188.0797	189.0875	189.0856	7.05	86.0966; **y (132.0663)**; $[M-COOH]^+$ (143.1175)
	Ala-Hyp	202.0954	203.1032	203.1051	-9.35	86.0966; **y (132.0667)**; $[M-CH_4N]^+$ (157.0979)
	Ser-Hyp	218.0903	219.0981	219.0956	8.41	86.0966; **y (132.0651)**; $[M-CH_5NO]^+$ (173.0954)
	Pro-Hyp	228.1110	229.1188	229.1160	6.22	70.0655; 86.0966; **y (132.0654)**
	Leu-Hyp	244.1059	245.1137	245.1150	-5.30	86.0966; **y (132.0654)**; $[M-COOH]^+$ (199.1069)
	Phe-Hyp	278.1267	279.1345	279.1380	-9.54	86.0966; b-CO(120.0815);**y (132.0657)**
Gly-Yaa-Hyp	Gly-Hyp-Pro	285.1325	286.1430	286.1403	9.4	y_2 (229.1538); b_2 (155.0810); **b_2-CO (127.0863)**
	Gly-Hyp-Phe	335.1481	336.1571	336.1559	3.5	y_1 (165.1254); y_2-NH_3 (253.1156); b_2 (171.1122); **b_2-CO (143.1173)**
	Gly-Hyp-Leu	301.1638	302.1699	302.1716	-5.6	y_1 (132.0662); b_2 (171.1122); **b_2-CO (143.1175)**

Table S4. Calibration curves, R^2, LOD, and LOQ for hydroxyproline-containing characteristic di- and tripeptides.

Peptide Category	Calibration curve	R^2	Linear range (μg/mL)	LOD (μg/kg)	LOQ (μg/kg)
Hyp-Yaa	y = 566.64x + 896.6	0.9999	0.01-200	1	5
Gly-Hyp-Yaa	y = 10.428x + 32.969	0.9983	0.05-200	15	50

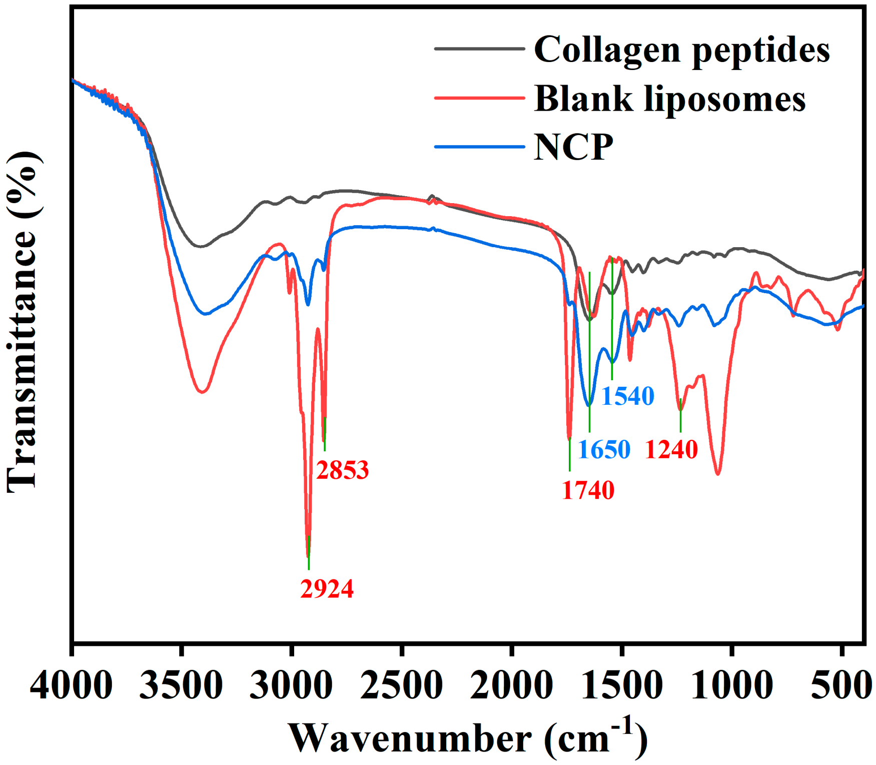

Figure S1. FT-IR spectra of collagen peptides (CP), blank nanoliposomes, and collagen peptide-loaded nanoliposomes (NCP).

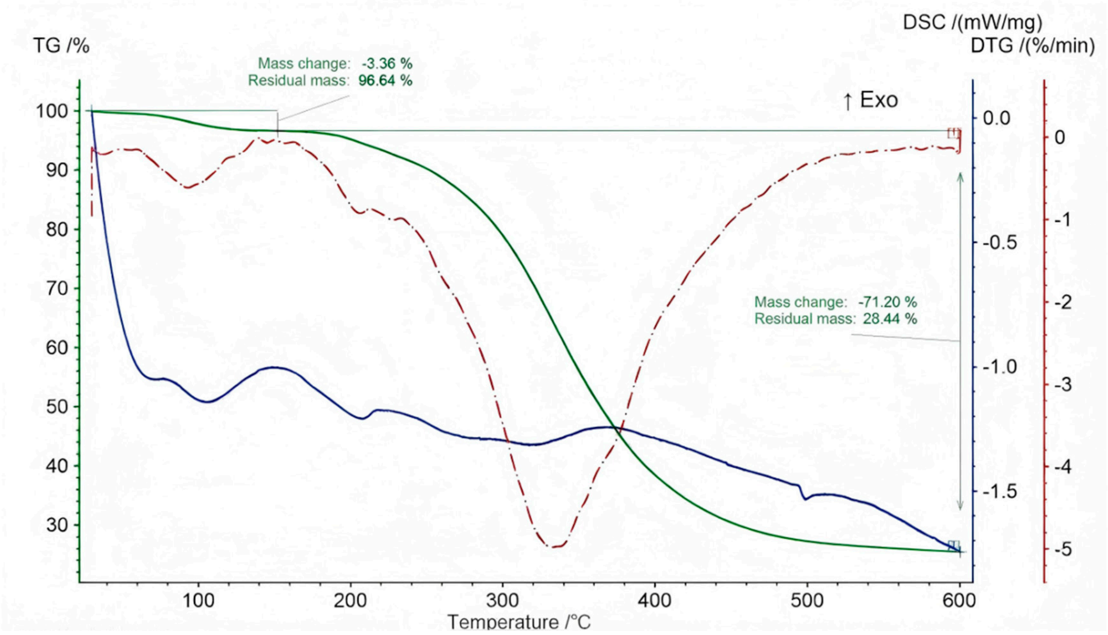

Figure S2. Thermogravimetric (TG), derivative thermogravimetric (DTG), and differential scanning calorimetry (DSC) curves of NCP.

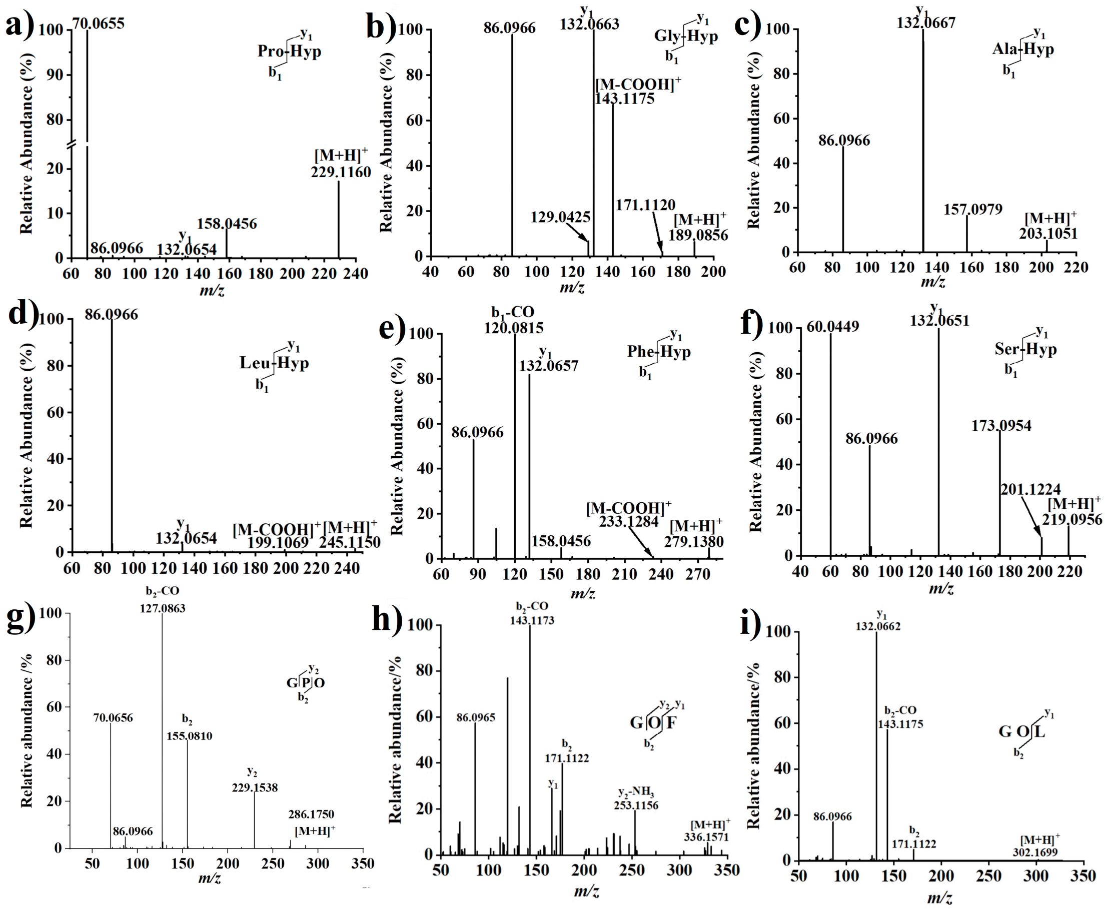

Figure S3. MS/MS spectra of Xaa-Hyp-type di/tripeptides in tilapia scale collagen peptides and NCP. (a) Pro-Hyp; (b) Gly-Hyp; (c) Ala-Hyp; (d) Leu-Hyp; (e) Phe-Hyp; (f) Ser-Hyp; (g) Gly-Pro-Hyp; (h) Gly-Hyp-Phe; (i) Gly-Hyp-Leu.
